# Associations of Adverse Childhood Experiences, Social Factors, and Mental Health in All-Cause Caregivers

**DOI:** 10.1093/geroni/igaf122.4074

**Published:** 2025-12-31

**Authors:** Maizonne Fields, Olivio Clay

**Affiliations:** The University of Alabama at Birmingham, Birmingham, Alabama, United States; The University of Alabama at Birmingham, Birmingham, Alabama, United States

## Abstract

The number of individuals needing assistance with personal care and household tasks from an informal caregiver is rising. Previous studies have also identified relationships between adverse childhood experiences (ACEs)—traumatic events related to violence, abuse, and/or exposure to mental health or substance use problems in others in the home— and mental health status. Using a life course perspective on aging, it is possible that experiencing could worsen mental health those that eventually served as a caregiver. This study uses data from the 2023 main wave of the Behavioral Risk Factor Surveillance System and includes all-cause caregivers. The study investigates the impact of living with someone that was depressed, mentally ill, or suicidal and being physically harmed (outside of spanking) more than once on the frequency of poor mental health days. Final analyses included 1180 participants that resided in either Oregon or Tennessee, 58.7% were females, and 37.4% being aged 65 and older. Linear multiple regression models revealed that living with someone that was depressed, mentally ill, or suicidal prior to age 18 (B=.174, p<.001) served as a better predictor of mental health than being physically harmed (outside of spanking) more than once (B=.094, p<.001). These models controlled for a series of demographic and social factors. Results suggest experiencing ACEs has the potential to worsen mental health in all-cause caregivers. These findings have implications for mental health providers and policymakers. Finally, results can inform researchers attempting to develop interventions for all-cause caregivers.

